# Hereditary angioedema in Latin America: international recommendations, expert consensus, and reported implementation barriers

**DOI:** 10.3389/fphar.2026.1933477

**Published:** 2026-10-01

**Authors:** Guillermo Arturo Guidos-Fogelbach, Andrea Aida Velasco-Medina, Freya Helena Campos-Romero, Nora Alarcón, Antonio Albarrán-Godínez, Mariana Bonifacino, Oscar Calderón, Iván Cherrez-Ojeda, Herberto José Chong-Neto, Gonzalo Chorzepa, Timothy Craig, Libia Susana Diez, Juan Carlos Fernández de Córdova-Aguirre, Ana María Gallardo, Masumi Grau-Tanabe, Maria-Rosario Grauert, Anete S Grumach, Dario Josviack, José Jesus López-Tiro, Ileana María Madrigal-Beas, Eli Mansour, Juan José Matta-Campos, Edison Morales, Blanca María del Refugio Morfin-Maciel, Sandra Agustina Nieto-Martínez, Margarita Olivares, Eduardo Enrique Piñeyro-Beltrán, Germán Darío Ramón, Jorge Mario Sánchez-Caraballo, Mauricio Sarrazola-Sanjuan, Joselit Torres, María Eugenia Vargas-Camaño, Daniel Vázquez, Solange Valle, Ricardo Zwiener

**Affiliations:** 1 Instituto Politécnico Nacional, SEPI/ENMH, Mexico City, Mexico; 2 Allergy and Clinical Immunology Department, Hospital General de México “Dr. Eduardo Liceaga”, Mexico City, Mexico; 3 Hospital Central Sur de Alta Especialidad, Mexico City, Mexico; 4 Hospital General IEESS Portoviejo, Manabí, Ecuador; 5 Unidad Integral de Alergia, Montevideo, Uruguay; 6 Clínica SANNA el Golf de San Isidro, Lima, Peru; 7 Universidad Espíritu Santo, Samborondón, Ecuador; 8 Respiralab Research Group, Guayaquil, Ecuador; 9 Charité – University Medical Center Berlin, a corporate member of Freie Universität Berlin and Humboldt‐Universität zu Berlin, Institute of Allergology, Berlin, Germany; 10 Fraunhofer Institute for Translational Medicine and Pharmacology ITMP, Immunology and Allergology, Berlin, Germany; 11 Universidade Federal do Parana Hospital de Clinicas, Curitiba, Brazil; 12 Sanatorio Parque, Rosario, Argentina; 13 Division of Pulmonary, Allergy and Critical Care Medicine, University of Pennsylvania, Philadelphia, PA, United States; 14 Universidad de Antioquia, Medellín, Colombia; 15 Hospital del Río, Cuenca, Ecuador; 16 Clínica Universidad de los Andes, Las Condes, Chile; 17 Clínica Alemana, Santiago, Chile; 18 Hospital Central de las Fuerzas Armadas, Montevideo, Uruguay; 19 Clinical Immunology, Centro Universitário Faculdade de Medicina ABC, Santo André, Brazil; 20 Instituto de Medicina Respiratoria, SantaFé, Argentina; 21 Hospital Regional Licenciado Adolfo López Mateos, Mexico City, Mexico; 22 UMAE Hospital de Especialidades del CMNO, IMSS, Guadalajara, Mexico; 23 Allergy and Immunology, Department of Internal Medicine, School of Medical Scieces, University of Campinas/UNICAMP, Campinas, Brazil; 24 Centro Médico Nacional, SXXI, IMSS, Mexico City, Mexico; 25 Unidad Alergologica, Medellin, Colombia; 26 Hospital San Ángel Inn, Mexico City, Mexico; 27 Instituto Nacional de Pediatría, Mexico City, Mexico; 28 Clinica de ISSSTE Especialidades Dr. Pedro Barcena Hiriart, San Luis Potosi, Mexico; 29 Hospital Italiano Regional del Sur, Bahía Blanca, Buenos Aires, Argentina; 30 Clinic “IPS Universitaria”, University of Antioquia, Medellin, Colombia; 31 Departamento de Medicina, Grupo GIPPAM, Universidad de Pamplona, Cúcuta, Colombia; 32 Asthma, Allergy and Immunology, Vargas Hospital Caracas, Caracas, Venezuela; 33 Centro Medico Nacional 20 de Noviembre, Mexico City, Mexico; 34 Diagnóstico Meeks, Buenos Aires, Argentina; 35 Hospital Universitário Clementino Fraga Filho Universidade Federal do Rio de Janeiro, Rio de Janeiro, Brazil; 36 Servicio de Alergia e Inmunología Clínica, Hospital Universitario Austral, Pilar, Buenos Aires, Argentina

**Keywords:** access to medicines, Delphi method, diagnosis, guideline implementation, hereditary angioedema, Latin America, long-term prophylaxis, on-demand therapy

## Abstract

**Background:**

Current international hereditary angioedema (HAE) guidelines and treat-to-target recommendations emphasize accurate diagnosis, early on-demand treatment, procedure-related short-term prophylaxis, individualized long-term prophylaxis (LTP), and routine assessment of disease control and quality of life. Evidence on implementation in Latin America remains limited. We aimed to assess concordance between international guidance and the consensus of a selected Latin American expert panel and to identify expert-reported implementation barriers.

**Methods:**

A scientific committee developed and externally validated a 74-item questionnaire (domains summarized in Supplementary Table S1) and conducted a two-round Delphi process among Latin American HAE experts. Consensus items were rated on a nine-point agreement scale, with consensus defined *a priori* as at least two-thirds of respondents scoring within a three-point band in the agreement or disagreement range. Descriptive items captured expert-reported diagnostic testing, treatment patterns, monitoring practices, and perceived implementation determinants. Consensus findings are summarized as meeting the prespecified threshold; descriptive findings are reported with item-specific n/N when available. Instrument validation (July-November 2024) showed high inter-rater agreement (Kendall W = 0.84; p < 0.001) and excellent internal consistency (Cronbach alpha = 0.92).

**Results:**

Thirty experts from 10 countries completed the process (Argentina, Brazil, Chile, Colombia, Ecuador, Mexico, Panama, Peru, Uruguay, and Venezuela). The prespecified consensus threshold was met for core biochemical diagnostic work-up, selective genetic evaluation, first-line targeted LTP (pdC1-INH, lanadelumab, and berotralstat), continued availability of on-demand therapy, routine monitoring, and management of special populations. The principal expert-reported implementation signal was limited feasibility: frequent or constant prophylaxis-supply interruptions were reported by 19/30 experts (63%), including 13/23 (57%) practicing in settings where public or insurance coverage was reported. Cost (19/30; 63%) and medicine availability (8/30; 27%) were the most frequently reported barriers. These reports were not independently verified against reimbursement, procurement, formulary, or inventory data.

**Conclusion:**

Within this selected panel, expert consensus was broadly concordant with international HAE guidance. Panelists reported barriers involving diagnostic testing, financing, medicine availability, and continuity of supply; however, these findings are perceptions and reported practices, not representative national or regional estimates or audited health-system data. They identify candidate implementation priorities that require confirmation through patient-level registries and objective access data.

## 1 Introduction

Hereditary angioedema (HAE) is a rare, potentially life-threatening bradykinin-mediated disorder characterized by recurrent, unpredictable episodes of subcutaneous and/or submucosal swelling affecting the skin, gastrointestinal tract, and upper airway (Busse and Christiansen, 2020; Busse et al., 2021; Maurer et al., 2022; Caballero et al., 2023; Vargas Camaño et al., 2023; Craig et al., 2026). Most cases are caused by C1 inhibitor (C1-INH) deficiency or dysfunction (HAE-C1-INH), whereas HAE with normal C1-INH comprises genetically heterogeneous entities with evolving diagnostic criteria (Maurer et al., 2022). A 2026 systematic review estimated a pooled minimal diagnosed annual prevalence of 1.70 per 100,000 across combined HAE types; only one South American study contributed, so a robust pooled prevalence estimate for Latin America is not currently available (Craig et al., 2026).

Latin American population-level data remain sparse. A Mexican mixed-methods analysis estimated an HAE prevalence of 0.9 per 50,000 inhabitants in 2019 and reported that cases recognized and treated in the national health system represented only 16.6% of the estimated number. This finding supports substantial underdiagnosis in Mexico, but the magnitude of underdiagnosis across Latin America cannot yet be estimated reliably (Nieto et al., 2023).

Clinical expression varies between and within patients. In the same Mexican analysis, cutaneous edema was reported in 67.5% of patients, abdominal pain in 47.9%, and a severe episode with laryngeal edema in 27.5%. Peripheral and gastrointestinal attacks are major sources of pain and disability, whereas upper-airway attacks require particular emphasis because they can rapidly cause fatal obstruction. Prodromal symptoms may include fatigue, numbness, tingling, and erythema marginatum (Jean-Baptiste et al., 2022; Leibovich-Nassi and Reshef, 2021; Nieto et al., 2023). Commonly reported triggers include trauma, stress, infection, and hormonal factors (Busse and Christiansen, 2020; Maurer et al., 2022).

Delayed diagnosis remains common and clinically consequential. Beyond the risk of asphyxiation from laryngeal attacks, prolonged diagnostic journeys amplify disease burden through repeated emergency visits, avoidable procedures, and psychosocial morbidity. In a patient-journey survey in Mexico, the mean time from symptom onset to diagnosis was 20 years and clinically relevant anxiety and depression were frequent, underscoring the patient-level impact of system delays in one Latin American setting (Vargas Camaño et al., 2023).

International guidelines increasingly define the goal of care as total disease control and the “normalization” of daily life, supported by treat-to-target frameworks that integrate attack prevention, early treatment of breakthrough attacks, and routine measurement of disease control and health-related quality of life (HRQoL). The WAO/EAACI 2021 revision and the US HAEA 2020 guideline recommend that long-term prophylaxis (LTP) be considered for all patients and individualized according to disease activity, attack burden, HRQoL, and patient preferences; they prioritize targeted agents (plasma-derived C1-INH replacement, Lanadelumab, Berotralstat) and restrict attenuated androgens or antifibrinolytics to settings where preferred options are unavailable or unsuitable (Maurer et al., 2022; Busse et al., 2021; Caballero et al., 2023; Vázquez et al., 2026).

However, prophylaxis does not eliminate risk. A 2024 systematic review of attacks occurring during LTP across multiple agents reported attack-free rates of approximately 40% with subcutaneous plasma-derived C1-INH at 16 weeks and 44% with lanadelumab 300 mg every 2 weeks at 6 months (77% during steady state), with on-demand therapy used in 49%–94% of breakthrough attacks and laryngeal events reported in 2%–7% of attacks (Longhurst et al., 2024). These findings reinforce that effective on-demand therapy (ODT) remains essential even for patients receiving LTP. Pivotal trials have demonstrated clinically meaningful reductions in attack frequency with subcutaneous pdC1-INH, lanadelumab, and once-daily oral berotralstat, providing the evidence base for guideline-preferred prophylaxis (Longhurst et al., 2017; Banerji et al., 2018; Zuraw et al., 2021; Wedner et al., 2021).

Even in high-resource settings, timely ODT use is inconsistent. In a US patient survey, 63.6% reported that they did not carry ODT at all times when away from home and 86% reported delaying treatment despite recognizing attack onset; when ODT was delayed, 75% experienced attacks that progressed in severity and 80% reported longer recovery (Betschel et al., 2024). In another survey, HRQoL and work productivity were substantially impaired during attacks, with worse EQ-5D-5L scores and greater work impairment as time to ODT increased, supporting guideline recommendations to treat early and to address practical barriers to prompt ODT administration (O’Connor et al., 2025).

At a global level, implementation of guideline-based care is inconsistent and strongly shaped by access and reimbursement. In the MENTALIST international physician survey, 48% of respondents reported that fewer than half of their patients had achieved WAO/EAACI treatment goals, and the highest-priority challenges were gaps in non-expert physician knowledge, treatment costs, and reimbursement for LTP (Buttgereit et al., 2025). Recent correspondence has further emphasized that, for many low- and middle-income countries, HAE-specific medications remain unavailable or inaccessible, necessitating equity-focused strategies beyond guideline publication (Jindal and Li, 2025). Real-world translation barriers are multifactorial: a qualitative study of US allergy/immunology clinicians identified insurance prior authorization, laboratory testing difficulties, time constraints, and deficits in physician and patient understanding as key barriers, while route/frequency of administration, medication samples, and shared decision-making were perceived as facilitators; notably, none of the clinicians used validated instruments to measure HAE-related QoL (Langmack et al., 2025).

Therapeutic burden and social determinants of health influence both treatment choice and outcomes. Reviews of HAE therapeutics highlight that oral preventive options may address practical needs and reduce injection-related barriers associated with parenteral prophylaxis and ODT, while future gene-targeted approaches may further reshape prevention strategies (Zanichelli et al., 2025). Disparities in diagnosis and management have been reported within diverse healthcare systems, including reduced diagnosis rates among Hispanic patients and historical underrepresentation of marginalized groups in clinical trials (Trickett et al., 2025). Strategies proposed to improve care in geographically underserved settings include earlier recognition, individualized management plans, telemedicine, and reliable access to ODT and prophylaxis (Riedl et al., 2022). A centralized comprehensive-care model has been associated with comparable disease control among rural vs. urban patients, suggesting a scalable approach for regions with limited specialist density (Holmes et al., 2024). Finally, experiences from Japan and China illustrate that national recommendations often evolve alongside local access to diagnostics and approved therapies, reinforcing the need to contextualize international guidance for region-specific realities (Hide et al., 2021; Xu et al., 2026).

Against this backdrop, we conducted a Latin American Delphi process to assess consensus on HAE diagnosis and comprehensive management and to document expert-reported implementation constraints. The analysis covers diagnostic work-up, on-demand treatment, short-term and long-term prophylaxis, monitoring, and care in special populations. It provides descriptive signals from participating experts regarding financing pathways, diagnostic testing, on-demand readiness, and continuity of supply. Because the panel was small and unevenly distributed across countries and access reports were not independently verified, the study is framed as a hypothesis-generating expert-consensus and implementation-perception analysis rather than a measurement of regional practice.

## 2 Materials and methods

Design and reporting framework. This expert-consensus study used a structured two-round Delphi methodology to examine concordance between international HAE guidance and the consensus and reported experience of a selected Latin American panel. The scope included diagnostic pathways, on-demand treatment, short-term prophylaxis, LTP, monitoring with patient-reported outcome tools, and perceived implementation determinants such as financing pathways, reimbursement, procurement, and continuity of supply. The study was not designed to estimate national or regional practice prevalence or to audit health-system access. Reporting was informed by established Delphi conduct and reporting recommendations (CREDES) (Jünger et al., 2017).

### 2.1 Panel recruitment, eligibility, and conflict-of-interest management

Eligible panelists were Latin American clinicians with documented experience in the diagnosis and management of HAE, including adult allergists/immunologists and pediatric immunology specialists. Non-specialists (e.g., primary care physicians) were excluded. Panelists were invited based on demonstrated clinical leadership and/or participation in regional angioedema networks. Thirty-three experts initiated participation; three were excluded because they did not meet the eligibility criteria or did not participate beyond round 1, leaving 30 experts for analysis. Potential conflicts of interest were managed prospectively. Panelists were asked to disclose relevant conflicts of interest in writing as part of study participation; these disclosures are reported in the Declarations section. Potential commercial influence was mitigated by developing the questionnaire on the basis of contemporary international guidelines and published evidence, conducting external validation by independent Latin American HAE specialists who were not members of the scientific committee, anonymizing Delphi voting, prespecifying consensus thresholds and analytical procedures, and reporting responses in aggregate. Therapeutic options were included and evaluated on the basis of published evidence, international recommendations, and panel responses. Details regarding the funding source and the role of the funder are provided in the Funding section.

### 2.2 Questionnaire development and structure

A scientific committee reviewed contemporary evidence and international recommendations and drafted a 74-item questionnaire covering: (i) clinical decision-making aligned with treat-to-target care (diagnostic approach and testing; attack recognition and early on-demand treatment; short-term prophylaxis; LTP initiation, switching, and management in special populations) and (ii) perceived implementation determinants (financing, reimbursement, access barriers, procurement, continuity of supply, and applicability of international guidance). Supplementary Table S1 reports the domains, item counts, representative content, and whether items were removed after content-validity review.

### 2.3 Content validation, item refinement, and reliability assessment

Internal and external validation were conducted between July and November 2024. The core scientific committee reviewed each item for semantic clarity, clinical relevance, and thematic coverage. Two independent panels of Latin American angioedema specialists, separate from the scientific committee, then assessed interpretability and feasibility in regional health-system contexts and provided quantitative ratings and qualitative comments. Relevance and clarity were rated on a four-point Likert scale, and the content validity index (CVI) was used to confirm prespecified thresholds (e.g., CVI ≥0.78) (Polit and Beck, 2006; Lynn, 1986). Qualitative feedback prompted wording revisions; all 74 items met the criteria after revision, and no item was removed after CVI filtering. Inter-group agreement was high (Kendall W = 0.84; p < 0.001), internal consistency was excellent (Cronbach alpha = 0.92), and per-item agreement ranged from 80% to 93%.

### 2.4 Delphi administration, scoring, and consensus criteria

Two online Delphi rounds were performed. Panelists rated consensus items on a nine-point scale (1–3 disagreement, 4–6 uncertainty, 7–9 agreement). Consensus was defined as at least two-thirds of respondents’ ratings falling within a three-point band in the agreement or disagreement range. Items not reaching consensus in round 1 were revised for wording or clarity in response to anonymized panel feedback and re-rated in round 2. Therapy-specific descriptive blocks used skip logic: panelists skipped therapies not used in their setting, so non-response may indicate non-use, lack of access, or limited experience rather than disagreement.

### 2.5 Data handling and analysis

Consensus findings are reported as meeting or not meeting the prespecified threshold. The denominator for each item was the number of panelists who responded to that item; where item-specific consensus n/N could not be verified from the aggregated report, standalone consensus percentages are omitted to avoid false precision. Descriptive findings are reported with item-specific counts and percentages whenever available, and missing responses were not imputed. Therapy-specific tables state their applicable N and label the combined category as “not prescribed/unknown” when skip logic prevents distinction between non-use and missingness. Country-level patterns are treated as illustrative reports from participating experts, not national estimates. The analysis did not independently verify perceptions against official reimbursement rules, formularies, procurement contracts, or inventory data. The manuscript is based on aggregated findings from the Latin American Delphi consensus report (October 2025) (Delphi Scientific Committee, 2025).

### 2.6 Ethics

This Delphi exercise captured expert opinion without patient-level data. Participation was voluntary, and responses were anonymized for aggregate reporting.

## 3 Results

Thirty experts from 10 Latin American countries completed the Delphi process: Argentina (n = 4), Brazil (n = 3), Chile (n = 2), Colombia (n = 5), Ecuador (n = 3), Mexico (n = 8), Panama (n = 1), Peru (n = 1), Uruguay (n = 2), and Venezuela (n = 1). Twenty-nine panelists were trained in Immunology-Allergology and one in Pediatric Immunology.

Panelists estimated that HAE type 1 accounted for 77% of cases, type 2 for 14%, and HAE with normal C1 inhibitor for 8% on average; the prespecified consensus threshold was met for type 1 being the most frequent. Adults aged 18–64 years were the predominant age group in the reported practices (mean 74% of each expert’s practice).

### 3.1 Diagnosis and comorbidities

The prespecified consensus threshold was met for combining C4 with C1-INH antigenic and functional assays to maximize diagnostic accuracy. Routine genetic testing was not supported for clearly established HAE-C1-INH; selective genetic evaluation was supported for uncertain diagnoses or variant characterization. Expert-reported testing patterns varied across participating experts and countries (Table 1).

**TABLE 1:** Expert-reported laboratory tests used for HAE diagnosis, by country (from Delphi report).

Country	Experts (n)	C1-INH antigen (n)	C1-INH function (n)	C4 mentioned (n)	Genetic testing
Argentina	4	1	3	—	—
Brasil	3	3	2	1	2
Chile	2	2	2	—	2
Colombia	5	4	1	—	​
Ecuador	3	3	​	—	1
México	8	6	3	1	—
Panamá	1	1	1	1	Occasionally
Perú	1	1	1	1	Occasionally
Uruguay	2	1	2	—	1
Venezuela	1	1	—	—	—

C4 was captured under “Other” in the original instrument and may therefore be underreported. “Occasionally” indicates non-routine genetic testing.

Most panelists reported clinically relevant comorbidities among their HAE patients (26/30; 87%). Among respondents reporting comorbidities (n = 26), the most frequently reported categories were arterial hypertension (69%), type 2 diabetes mellitus (46%), anxiety/depression (42%), and autoimmune disease (27%).

Based on a prioritization exercise, the highest-priority criteria for initiating long-term prophylaxis (LTP) were monthly attack frequency (priority 10), followed by attack severity, quality of life, and attack location (priority 9). Patient age and pregnancy/lactation status were also considered important (priority 8).

The highest-priority criteria for switching LTP were failure to reduce or an increase in attack frequency (priority 10), followed by lack of improvement in attack severity, adverse events, and increasing HAE symptoms (priority 9). Marked quality-of-life impairment and medication availability were additional drivers (priority 8).

### 3.2 Therapeutic management: prophylaxis within comprehensive care

The prespecified consensus threshold was met for pdC1-INH, lanadelumab, and berotralstat as first-line LTP options, consistent with international guidance. Consensus was also reached that serious adverse events with these agents are rare to very rare. Attenuated androgens were positioned as alternatives when preferred targeted therapies are unavailable, unsuitable, or infeasible, and antifibrinolytics as lower-line options, with selective consideration in children or pregnancy when targeted options are inaccessible. Effective on-demand therapy should remain available to all patients, including those receiving prophylaxis.

Expert-reported preventive treatment patterns. Participating experts described marked heterogeneity in therapies used as first-line prevention and in prescribing frequency by agent (Tables 2, 3). Because therapy-specific blocks used skip logic, apparent non-prescription should be interpreted cautiously: non-response may reflect restricted availability or limited experience rather than a negative clinical preference.

**TABLE 2:** Treatments reported as first-line long-term prophylaxis in practice, by country (from Delphi report).

Country	pdC1-INH mentions	Lanadelu-mab mentions	Berotralstat mentions	Antifibrino-lytics mentions	Attenuated androgens mentions
Argentina	2	4	4	—	1
Brasil	1	3	—	1	2
Chile	2	—	—	—	1
Colombia	—	5	—	1	3
Ecuador	—	—	—	1	2
México	1	5	—	—	—
Panamá	—	—	—	1	1
Perú	—	—	—	1	—
Uruguay	—	Vía litigio	Vía litigio	2	1
Venezuela	—	—	—	1	—

Counts indicate how many times each therapy was mentioned in expert free-text responses within each country; experts could report more than one first-line option. These data illustrate heterogeneity among participating experts and should not be interpreted as national estimates. Some mentions may reflect episodic access (e.g., litigation-based access) rather than routine reimbursement or procurement. The number of participating experts per country is provided in Table 4.

**TABLE 3:** Overall frequency of prescribing long-term prophylaxis options (from Delphi report, N = 30 experts).

Frequency category	pdC1-INH (n)	Lanadelumab (n)	Berotralstat (n)	Androgens (n)	Antifibrinolytics (n)
Not prescribed/unknown	17	9	24	16	17
Rarely	7	2	1	3	2
Sometimes	5	5	3	6	5
Frequently	1	9	2	5	1
Always	0	5	0	0	5

“Not prescribed/unknown” combines explicit “never” and missing responses generated by skip logic for therapies not prescribed in the expert’s setting. Accordingly, this category may reflect lack of access or limited experience rather than a negative clinical preference. Counts sum to N = 30 experts per therapy.

Expert-reported access, reimbursement, and availability. Participating experts described heterogeneous public coverage, private insurance, and out-of-pocket pathways (Table 4). Cost (19/30; 63%) and medicine availability (8/30; 27%) were the most frequently reported barriers to guideline-preferred prophylaxis. Frequent or constant supply interruptions were reported by 19/30 experts (63%), including 13/23 (57%) who reported practicing in settings with public or insurance coverage. These findings reflect panelist reports and were not independently verified against official reimbursement policies, procurement records, formularies, or pharmacy inventories.

**TABLE 4:** Country-level reimbursement, availability, and guideline applicability (from Delphi report).

Country	Experts (n)	Predominant funding (panel)	Public coverage (majority)	Availability issues (freq/always, %)	Main access barrier	Guideline applicability
Argentina	4	Insurance	Yes	75	Costs	Full
Brasil	3	Mixed	No	100	Costs	Low/Moderate/Full
Chile	2	Self-funded	No	100	Costs	Low/Moderate
Colombia	5	Insurance	Yes	0	Costs/drug availability	Moderate/High
Ecuador	3	Self-funded	No	67	Costs/drug availability	None/Low
México	8	State	Yes	50	Costs	High
Panamá	1	Mixed	Yes	100	Costs	Low
Perú	1	Self-funded	No	100	Drug availability	High
Uruguay	2	State/Mixed	Yes	100	Costs/drug availability	Low/Moderate
Venezuela	1	Self-funded	No	100	Drug availability	Low

Values reflect responses from participating experts and may not represent national policy, reimbursement rules, or population-level access. Public coverage reflects the majority response within each country’s small expert sample. Access through exceptional routes (e.g., litigation) may not represent routine procurement. ‘Availability issues’ denotes experts reporting frequent or constant access/supply problems. No response was independently validated against official policy, procurement, formulary, or inventory data.

Country-level tables are included only to illustrate heterogeneity in the participating panel. Per-country samples were small and uneven (often n = 1–3), so country-specific distributions should be interpreted as illustrative expert reports, not estimates of national policy, population-level access, treatment frequency, or quality of care. Cross-country comparisons are therefore descriptive and hypothesis-generating.

### 3.3 Monitoring and patient-reported outcomes

The prespecified consensus threshold was met for routine assessment of quality of life, disease activity, and disease control using validated instruments. Although 28/30 experts (93%) stated that they assess quality of life, only 18/30 (60%) named an instrument; not every named measure was a validated HAE-specific patient-reported outcome measure (Table 5). Thus, 18/30 is the more conservative estimate of structured PROM implementation. Expert-reported satisfaction was descriptively higher for targeted therapies than for antifibrinolytics or attenuated androgens, with therapy-specific denominators shown in Table 6.

**TABLE 5:** Quality-of-life instruments used in routine follow-up (from Delphi report).

Instrument	Mentions (total 24)	%
AE-QoL or HAE-QoL or HAQoL	13	54%
AECT	4	17%
QoL (unspecified)	2	8%
DLQI	2	8%
Number of attacksduring the period	1	4%
Clinical history only	1	4%
AAS	1	4%

Percentages are calculated among experts who named at least one instrument (18/30); 24 total mentions were recorded because respondents could name more than one instrument.

**TABLE 6:** Patient satisfaction with long-term prophylaxis therapies as reported by experts (from Delphi report).

Satisfaction level	pdC1-INH (N = 20)	Lanadelu-mab (N = 22)	Berotralstat (N = 13)	Antifibrino-lytics (N = 23)	Attenuated androgens (N = 27)
Very dissatisfied	0%	0%	0%	9%	7%
Dissatisfied	5%	0%	0%	17%	19%
Neutral	20%	5%	15%	39%	30%
Satisfied	65%	18%	23%	30%	44%
Very satisfied	10%	77%	62%	4%	0%

Percentages are calculated among experts responding for each therapy; N differs by therapy.

### 3.4 Special populations, short-term prophylaxis, and on-demand therapy

The prespecified consensus threshold was met for pdC1-INH as first-line LTP during pregnancy and lactation. In pediatric care, pdC1-INH and lanadelumab were preferred, with berotralstat mainly considered for adolescents aged 12 years and older. For procedure-related short-term prophylaxis, pdC1-INH was the most frequently cited option among responding experts (Table 7). Consensus was also reached on maintaining on-demand therapy during LTP; among experts who specified an agent, icatibant was most frequently cited.

**TABLE 7:** Short-term prophylaxis options used in practice by country (from Delphi report).

Country	pdC1-INH	Icatibant	Lanadelu-mab	Berotral-stat	Tranexamic acid	Other/no response
Argentina	4	—	—	—	—	—
Brasil	3	—	—	—	—	—
Chile	1	—	—	—	—	1
Colombia	4	1	—	—	—	—
Ecuador	—	—	1	1	—	1
México	3	3	1	—	—	1 (progestins and omalizumab)
Panamá	1	—	—	—	—	—
Perú	1	—	—	—	—	—
Uruguay	2	—	—	—	1	—
Venezuela	—	—	—	—	1	—

Entries reflect free-text expert responses and may include local terminology.

### 3.5 Perceived applicability of international guidelines

Among participating experts, 15/30 (50%) rated international guideline recommendations as highly or totally applicable to their daily practice (Table 8). The remainder reported moderate applicability (7/30; 23%), low applicability (6/30; 20%), or no applicability (1/30; 3%); one expert did not respond.

**TABLE 8:** Applicability of international guideline recommendations to local practice, by country (from Delphi report).

Country	None	Low	Moderate	High	Full
Argentina	0	0	0	0	4
Brasil	0	1	1	0	1
Chile	0	1	1	0	0
Colombia	0	0	2	2	1
Ecuador	1	1	0	0	0
México	0	0	2	4	2
Panamá	0	1	0	0	0
Perú	0	0	0	1	0
Uruguay	0	1	1	0	0
Venezuela	0	1	0	0	0

Counts represent number of experts selecting each applicability level.

Perceived changes in attack frequency and disease control after LTP initiation were reported as expert impressions rather than measured outcomes. Because the survey did not operationally define “disease control” and these items are susceptible to recall bias and access-driven case selection, these results are presented as qualitative signal indicators (Supplementary Tables S2, S3) and interpreted cautiously in the Section 4.

## 4 Discussion

### 4.1 Guideline concordance and the treat-to-target rationale

This Delphi exercise found broad alignment within the participating panel between expert consensus and international recommendations on diagnostic work-up, preferred LTP options, and treat-to-target goals (Maurer et al., 2022; Busse et al., 2021; Caballero et al., 2023). It does not estimate the frequency of these practices in Latin America. Its primary contribution is to juxtapose panel consensus with structured reports of implementation constraints. Financing, procurement, and continuity-of-supply findings should therefore be interpreted as hypothesis-generating perceptions rather than verified regional health-system measurements.

### 4.2 Access, reimbursement, and continuity of supply as dominant drivers of divergence

Participating experts most often reported cost, medicine availability, and continuity of supply as barriers to guideline-preferred prophylaxis. Frequent or constant supply interruptions were reported by 19/30 (63%), including 13/23 (57%) who reported public or insurance coverage. These observations are coherent with the MENTALIST survey and recent discussions of global disparities, which identified treatment cost, reimbursement, and non-expert knowledge as major unmet needs (Buttgereit et al., 2025; Jindal and Li, 2025). However, the present study did not verify coverage status, procurement performance, stock levels, or causal mechanisms. The findings therefore identify candidate explanations for implementation difficulty rather than establish objective country-level access conditions. Prior authorization, laboratory testing, time constraints, and knowledge gaps have also been documented in a US qualitative study (Langmack et al., 2025). In Latin America, exceptional access routes such as litigation may contribute to episodic availability, but this mechanism likewise requires country-specific verification.

### 4.3 Breakthrough attacks on prophylaxis and the imperative of on-demand readiness

A key point of convergence between international evidence and the panel is the need to maintain effective ODT for all patients, including those receiving LTP. A systematic review showed that breakthrough attacks occur across anatomic locations, including the larynx, and that ODT is used in 49%–94% of breakthrough attacks (Longhurst et al., 2024). The panel reached consensus on maintaining ODT during LTP. Several experts did not specify an available ODT agent; because skip logic and access were not independently assessed, this absence cannot be attributed to a single cause. Behavioral and practical barriers to early ODT are documented in a US patient survey: 63.6% did not carry ODT at all times away from home and 86% delayed treatment; among delayed attacks, 75% progressed in severity and 80% had longer recovery (Betschel et al., 2024).

Complementary patient-reported data show marked declines in HRQoL during attacks (mean EQ-5D-5L index 0.849 “today” vs. 0.568 “during the last treated attack”) and worse HRQoL and work impairment as time to ODT increases; overall work impairment in the week after attack onset approached 39% (O’Connor et al., 2025). These external data support evaluating interventions that improve reliable ODT availability, portability, and early-use education in Latin American settings. Their effect in the region should be measured prospectively rather than inferred from the present expert survey.

### 4.4 Treatment burden, route of administration, and implications for LTP selection

The panel’s consensus and reported prescribing patterns reveal a pragmatic tension between evidence-based preferences and access-shaped practice. Although targeted agents were endorsed as first-line options, participating experts reported continued use of attenuated androgens and antifibrinolytics in some settings. Berotralstat was endorsed but infrequently reported as prescribed. These patterns may reflect regulatory, coverage, distribution, patient-preference, or experience-related factors, but the survey cannot distinguish among them. Contemporary reviews emphasize that route and frequency of administration influence treatment burden and shared decision-making, while implementation research identifies administration logistics, time, and system processes as potential barriers or facilitators (Langmack et al., 2025; Zanichelli et al., 2025).

### 4.5 Monitoring and patient-reported outcomes: from expert endorsement to routine implementation

Treat-to-target care requires explicit targets and routine reassessment with validated instruments for disease control and HRQoL (Caballero et al., 2023). The distinction between recommendation and implementation was evident in this panel: 28/30 experts reported assessing QoL, but only 18/30 named an instrument, and some named approaches were not validated HAE-specific PROMs. The 18/30 figure is therefore a more conservative estimate of structured PROM use than the general self-report of assessment. A similar implementation gap has been described in a US qualitative study, in which none of the interviewed clinicians reported using a validated HAE-related QoL instrument (Langmack et al., 2025). Future questionnaire designs should separate a general question about assessment from verification items requiring the instrument name, administration frequency, and, where relevant, recall period. This approach may reduce recall-related or socially desirable overreporting. Pragmatic use of a small, standardized measure set could also improve clinical decisions and generate locally relevant evidence (O’Connor et al., 2025).

### 4.6 A pragmatic treat-to-target package for Latin America

As a hypothesis-generating implementation proposal rather than a validated regional standard, we outline a minimal treat-to-target package that is aligned with international guidance and could be tested across diverse Latin American settings (Caballero et al., 2023).

1. Disease control instrument: Angioedema Control Test (AECT) at baseline and every follow-up visit. Suggested target: AECT ≥10 indicates well-controlled disease, whereas AECT <10 suggests inadequate control and should prompt reassessment of adherence, access to on-demand treatment, and the prophylaxis plan. A change of ≥3 points can be considered clinically meaningful for monitoring response over time (Weller et al., 2020).
2. HRQoL instrument: AE-QoL or HAE-QoL (choose the locally available language version) at baseline and at least every 6–12 months, and after major treatment changes. Routine HRQoL measurement supports shared decision-making and can strengthen local health-technology and reimbursement discussions by documenting benefit beyond attack counts (Caballero et al., 2023).
3. Reassessment interval and triggers: reassess within 8–12 weeks after initiating or switching LTP, then every 3–6 months once stable. Trigger an earlier review after any laryngeal attack, emergency visit, or repeated late-treated/untreated attacks, and ensure that effective on-demand therapy is continuously available even when on LTP (Caballero et al., 2023; Longhurst et al., 2024; O’Connor et al., 2025).

### 4.7 Diagnostic delay, geographic inequity, and implementable care models for Latin America

Several panel findings, including consensus on standardized diagnostic testing and reported country-level variability, should be interpreted alongside patient-level evidence from the region. In Mexico, diagnostic delays of approximately 20 years and substantial psychosocial impact have been reported, suggesting that recognition, referral, and confirmatory testing may be major points of attrition before LTP is considered (Vargas Camaño et al., 2023). Geographic barriers and low specialist density can exacerbate delay; rural-care analyses support structured management plans, telemedicine, and reliable medication access (Riedl et al., 2022). A centralized comprehensive-care model achieved comparable angioedema control among rural and urban patients, suggesting that concentrated expertise and coordinated care may mitigate geographic inequity (Holmes et al., 2024). National consensus efforts in Japan and China further illustrate that international evidence can be adapted to local diagnostic and therapeutic availability (Hide et al., 2021; Xu et al., 2026).

### 4.8 Recent Latin American registry evidence and diagnostic complexity

Recent patient-level registries provide an important counterpoint to this expert survey. The Brazilian multicenter registry included 820 patients; 72.4% had HAE-C1-INH and 19.4% had HAE-FXII. Only 52.8% received first-line on-demand therapy and 4.8% received first-line targeted LTP, whereas 52% used attenuated androgens (Ferriani et al., 2025). In Argentina, a nationwide registry of 538 patients reported a median 10-year diagnostic delay, HAE-1 in 76%, F12 variants in 7%, DAB2IP variants in seven patients, attack-specific treatment in 88%, and LTP in 28.34% (Josviak et al., 2026). These patient-level findings are directionally consistent with concerns about diagnostic delay and uneven treatment access, but they do not validate country-level estimates from the Delphi panel because study designs, populations, and measures differ.

Together, these reports support phenotype-guided biochemical testing and selective genetic evaluation, particularly when C1-INH results are normal or the phenotype is atypical. A recent case report of concurrent HAE type I and common variable immunodeficiency, initially evaluated as suspected antibiotic hypersensitivity, illustrates the value of broader immunologic assessment when recurrent infections, hypogammaglobulinemia, poor vaccine responses, or other atypical features coexist (Urdea et al., 2026). Because this is a single case, it should be interpreted as a diagnostic reminder rather than evidence that HAE and common variable immunodeficiency commonly co-occur.

### 4.9 Contextualizing the panel against current international guidance

Current international HAE guidelines and consensus documents converge on standardized diagnosis with C4 and C1-INH antigenic and functional testing, selective genetics, early attack treatment and home readiness, continued ODT during prophylaxis, procedure-related pdC1-INH, individualized LTP, and comprehensive follow-up. The pediatric guideline extends these principles to age-specific care (Maurer et al., 2022; Busse et al., 2021; Caballero et al., 2023; Vázquez et al., 2026; Farkas et al., 2026). Against this framework, the participating panel showed broad directional concordance in clinical intent (Figure 1). This statement describes consensus within the selected panel and does not imply that the same practices are routinely implemented across Latin America.

**FIGURE 1:**
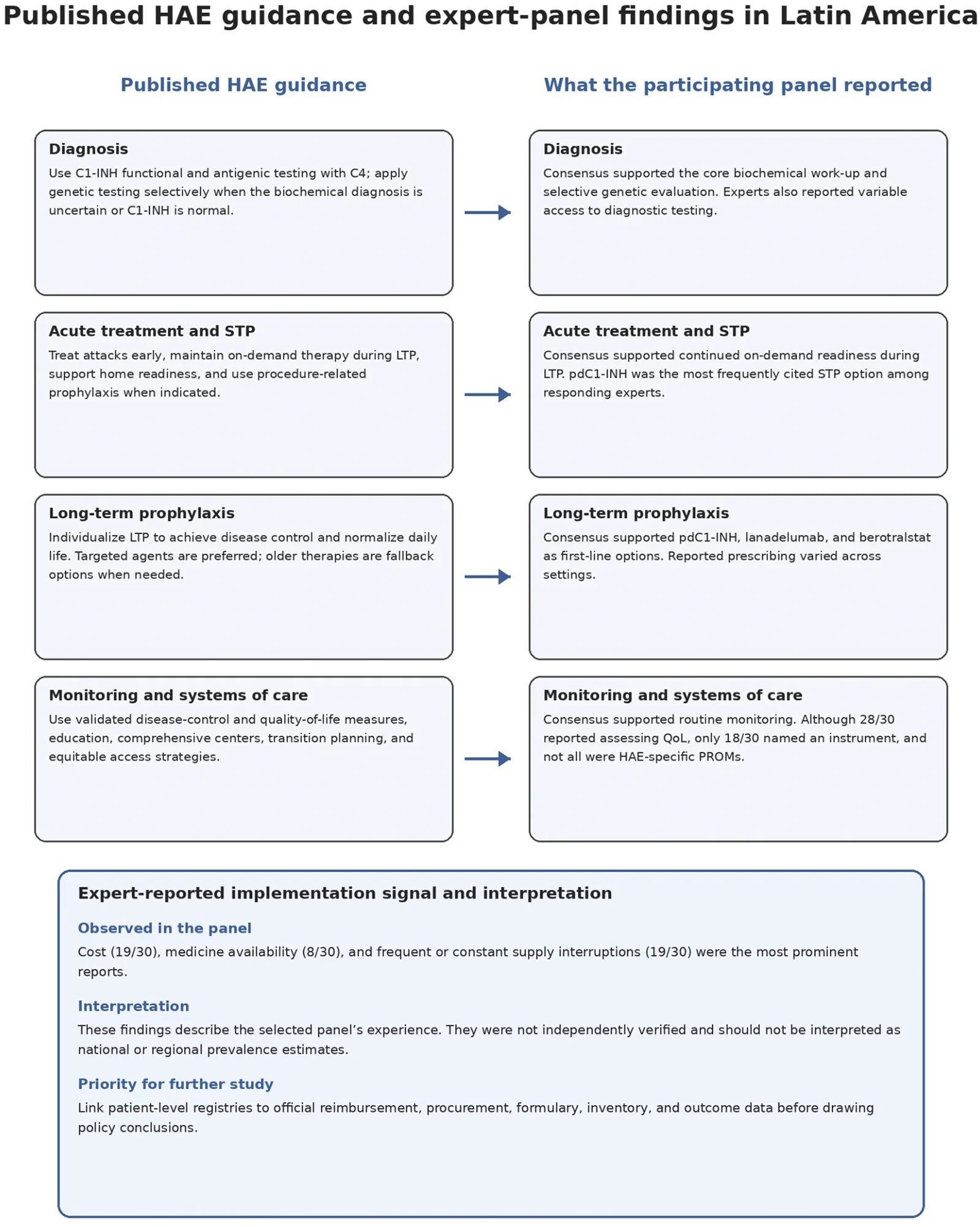
Published HAE guidance and expert-panel findings in Latin America. This conceptual figure contrasts guideline-defined priorities with the participating panel’s consensus and expert-reported implementation signals. Panel findings are not representative national or regional estimates, and reported access constraints were not independently verified (Maurer et al., 2022; Busse et al., 2021; Caballero et al., 2023; Vázquez et al., 2026; Farkas et al., 2026).

The relevant contrast in this study is between what international guidance recommends and what participating experts reported their settings could deliver. Cost and medicine availability were the most frequently reported barriers, and 19/30 experts reported frequent or constant supply interruptions, including 13/23 who reported public or insurance coverage. Read with the methodological caveats above, these data support an expert-reported implementation-feasibility signal rather than an objective regional implementation estimate (Figure 2). Laboratory access, financing, procurement, and distribution should be evaluated with country-specific policy, purchasing, inventory, and patient-level outcome data (Maurer et al., 2022; Busse et al., 2021; Caballero et al., 2023; Vázquez et al., 2026; Farkas et al., 2026).

**FIGURE 2:**
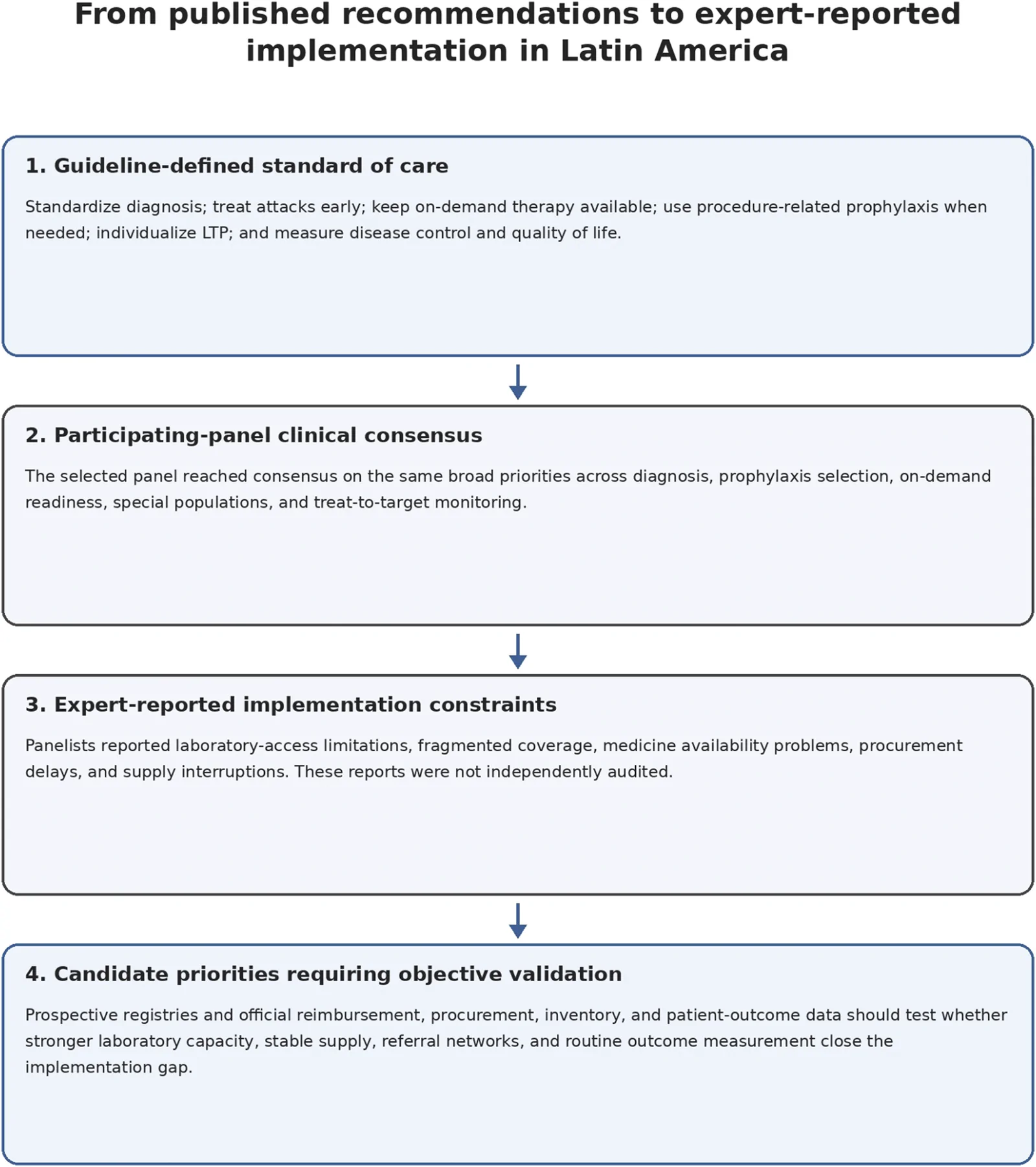
From published recommendations to expert-reported implementation in Latin America. This conceptual figure separates guideline standards, participating-panel consensus, reported constraints, and candidate operational priorities requiring objective validation (Farkas et al., 2026).

Within this selected panel, consensus favored guideline- concordant HAE management, while reported implementation constraints were prominent as shown in Table A1. Candidate priorities arising from these reports include stronger laboratory capacity, more stable procurement and distribution, simpler coverage pathways, referral networks, telemedicine, and routine outcome measurement. Their relevance and impact should be tested rather than assumed. Prospective registries should link verified access status, treatment patterns, and validated patient-reported outcomes; country-level studies should incorporate regulatory approvals, health-technology assessments, procurement contracts, and stock-continuity data.

### 4.10 Limitations

This study reports expert consensus and expert-reported practice rather than patient-level, prospectively measured outcomes. Per-country respondent numbers were small (often n = 1–3), and results should not be interpreted as national policy positions or population-level estimates of access or disease control. Several descriptive blocks used skip logic for therapies not prescribed in each setting; accordingly, apparent non-prescription can conflate true non-use with restricted availability or limited experience, and denominators vary by therapy and item. Perceived changes in attack frequency and “disease control” represent subjective impressions; because control was not operationally defined and these items are susceptible to recall bias and access-driven case selection, they should be interpreted as qualitative signals rather than comparative effectiveness evidence. The study was supported by an unrestricted grant from Takeda; although the funder had no role in questionnaire design, data analysis, or manuscript drafting, residual perception of sponsor alignment bias is possible and should be considered alongside the methodological safeguards (anonymized scoring, prespecified consensus rules, and independent external validation). Finally, the analysis did not incorporate drug-level regulatory status, formal health technology assessment outcomes, or procurement contract data in each country.

Additional limitations concern measurement transparency and external validity. The full item-level questionnaire and a round-specific wording-change log are not reproduced in this manuscript, limiting independent assessment of wording effects and exact replication of the Delphi process. Supplementary Table S1 provides domain-level item counts, representative content, and the result of CVI filtering, while the final questionnaire and available de-identified round-level documentation may be requested from the corresponding author. In addition, standalone consensus percentages were omitted when item-specific n/N could not be verified from the aggregated report. The access findings reflect panelist reports and were not audited against official reimbursement, formulary, procurement, contract, or inventory sources. Selection of recognized HAE experts may also overestimate guideline awareness compared with the broader clinician population.

## 5 Conclusion

Within this selected and unevenly distributed panel of 30 experts from 10 countries, the prespecified consensus threshold was met for core elements of evidence-based HAE diagnosis and comprehensive management. Panelists reported constraints involving diagnostic access, financing pathways, medicine availability, and supply continuity. Because these reports were not independently verified and several countries were represented by only one to three respondents, the findings should not be generalized as national or regional estimates. They identify candidate implementation priorities—laboratory capacity, administrative simplification, sustainable coverage, procurement resilience, and routine measurement of disease control and quality of life—that require validation with patient-level and objective health-system data.

## Data Availability

The original contributions presented in the study are included in the article/Supplementary Material, further inquiries can be directed to the corresponding author.

## Appendix A

**TABLE A1:** Alignment matrix between international guidelines, participating-panel consensus, and expert-reported implementation signals.

Domain	International guideline position	Participating-panel consensus	Expert-reported implementation signal
Diagnosis	C4 plus C1-INH antigenic and functional testing; selective genetic testing for uncertain cases and HAE with normal C1-INH (Maurer et al., 2022; Vázquez et al., 2026).	Consensus threshold met for combined biochemical diagnosis and selective genetic evaluation	Participating experts reported variation in test use; C4 may be underreported because it was captured under “other.”
First-line LTP	Preferred LTP options include pdC1-INH, lanadelumab, and berotralstat (Maurer et al., 2022; Busse et al., 2021; Caballero et al., 2023; Vázquez et al., 2026).	Consensus threshold met for pdC1-INH, lanadelumab, and berotralstat as first-line LTP.	Participating experts reported heterogeneous use; low reported use of some targeted agents may reflect access or experience, but was not objectively verified
Older therapies	Attenuated androgens and antifibrinolytics are lower-line options because of safety or efficacy limitations (Maurer et al., 2022; Busse et al., 2021; Caballero et al., 2023; Vázquez et al., 2026).	Consensus threshold met for positioning androgens as alternatives when preferred therapies are unavailable and antifibrinolytics as lower-line options	Participating experts reported continued use in some settings; the survey cannot determine whether access, cost, preference, or other factors drove this pattern
ODT availability	All patients should have effective on-demand therapy, including during prophylaxis (Maurer et al., 2022; Busse et al., 2021; Caballero et al., 2023; Vázquez et al., 2026).	Consensus threshold met for maintaining ODT during LTP.	Only a subset of experts specified an ODT agent; icatibant predominated among those responding, and skip logic limits interpretation
Monitoring	Assess disease activity, burden, control, and QoL with validated tools (Maurer et al., 2022; Caballero et al., 2023; Vázquez et al., 2026).	Consensus threshold met for routine QoL and disease-control assessment	28/30 reported assessing QoL, but only 18/30 named an instrument; not all named measures were validated HAE-specific PROMs
Special populations	pdC1-INH is preferred in pregnancy/lactation and is an important pediatric option; age and label constraints apply to other agents (Maurer et al., 2022; Busse et al., 2021; Vázquez et al., 2026; Ferriani et al., 2025).	Consensus threshold met for pdC1-INH in pregnancy/lactation and for pdC1-INH/lanadelumab in pediatric care	Participating experts most often cited pdC1-INH for STP; perceived feasibility varied with reported access

Consensus cells indicate that the prespecified threshold was met; standalone percentages are omitted where item-level n/N could not be verified. Implementation cells summarize reports from participating experts and should not be interpreted as objective national or regional data. Full guideline details are provided in the cited references.
