# Supplementary material for: Hereditary angioedema in Latin America: international recommendations, expert consensus, and reported implementation barriers
Source: Front Pharmacol. 2026 Oct 1;17:1933477. doi: 10.3389/fphar.2026.1933477 (PMC13668836; doi:10.3389/fphar.2026.1933477)
Supplement: Supplementary file 1 [file Table1.docx]

Supplementary Material

**Supplementary Table S1. Questionnaire domains and content-validation summary.**

| Domain | Items (n) | Representative content (examples) | Items removed after CVI filtering (n) |
| --- | --- | --- | --- |
| Epidemiology and clinical profile | 4 | HAE type distribution; age at onset; key clinical phenotypes | 0 |
| Diagnosis and testing | 7 | C4; C1-INH antigen/function; genetic testing indications; differential diagnosis | 0 |
| Comorbidities and patient profile | 4 | Comorbidities influencing LTP choice; contraindications; patient circumstances | 0 |
| LTP selection: preferred options and positioning | 11 | First-line options (pdC1-INH, Lanadelumab, Berotralstat); shared decision-making; escalation | 0 |
| Alternative/legacy prophylaxis | 6 | Attenuated androgens; antifibrinolytics; risk–benefit considerations | 0 |
| Access, reimbursement, and continuity of supply | 12 | Funding mechanisms; procurement; supply interruptions; administrative barriers; equity considerations | 0 |
| Prescribing patterns and on-demand readiness during LTP | 10 | Frequency of prescribing by therapy; ODT while on LTP; short-term prophylaxis practices | 0 |
| Perceived outcomes and patient satisfaction | 5 | Perceived attack reduction; perceived control; satisfaction; treatment burden | 0 |
| LTP initiation criteria | 5 | Attack frequency/severity; laryngeal risk; QoL impact; patient preference; access feasibility | 0 |
| LTP switching criteria | 5 | Breakthrough attacks; adverse effects; adherence; access/supply changes; patient goals | 0 |
| Monitoring and treat-to-target implementation | 5 | Disease control/QoL instruments; follow-up intervals; reassessment triggers | 0 |

Note: The initial draft comprised 74 items. All items met prespecified content-validity criteria after wording revisions, and no item was removed. This table provides domain-level transparency but does not reproduce the full item wording or round-specific change log.

**Supplementary Table S2. Expert-perceived change in attack frequency after initiation of long-term prophylaxis (from Delphi report).**

| Therapy (respondents) | Attack frequency decreased (Yes %) | Attack frequency decreased (No %) |
| --- | --- | --- |
| pdC1-INH (n=22) | 91 | 9 |
| Lanadelumab (n=23) | 91 | 9 |
| Berotralstat (n=16) | 81 | 19 |
| Antifibrinolytics (n=23) | 61 | 39 |
| Attenuated androgens (n=27) | 89 | 11 |

Note: Percentages are calculated among experts who responded for each therapy and reflect perceived change rather than measured outcomes.

**Supplementary Table S3. Expert-perceived level of disease control associated with observed changes in attack frequency (from Delphi report).**

| Therapy (respondents) | No control (%) | Low control (%) | Moderate control (%) | Well controlled (%) | Fully controlled (%) |
| --- | --- | --- | --- | --- | --- |
| pdC1-INH (n=22) | 0 | 0 | 14 | 64 | 23 |
| Lanadelumab (n=23) | 0 | 0 | 4 | 43 | 52 |
| Berotralstat (n=14) | 0 | 0 | 14 | 71 | 14 |
| Antifibrinolytics (n=21) | 14 | 29 | 52 | 5 | 0 |
| Attenuated androgens (n=27) | 0 | 11 | 63 | 26 | 0 |

Note: The survey did not operationally define 'disease control'; interpretation may vary by respondent. These data are qualitative signal indicators and should not be interpreted as comparative effectiveness evidence.
